# Angioplastia Pulmonar com Balão Percutâneo Sistemática com Estadiamento em Hipertensão Pulmonar Tromboembólica Crônica Inoperável Grave

**DOI:** 10.36660/abc.20190717

**Published:** 2021-02-02

**Authors:** Kazuhiro Dan, Akira Shionoda, Hiromi Matsubara

**Affiliations:** 1 Ichinomiya Nishi Hospital Ichinomiya Japão Ichinomiya Nishi Hospital, Ichinomiya - Japão; 2 Okayama Medical Center Okayama Japão Okayama Medical Center, Okayama - Japão

**Keywords:** Angioplastia Pulmonar com Balão, Hipertensão Pulmonar, Embolia Pulmonar, Fragilidade, Idosos

## Introdução

O tratamento para hipertensão pulmonar tromboembólica crônica (HPTEC) é limitado a endarterectomia pulmonar (PEA), terapia medicamentosa e angioplastia pulmonar percutânea com balão (APB).^1^ O tratamento padrão-ouro é a PEA, e pacientes com HPTEC e lesões proximais são geralmente bons candidatos cirúrgicos. Complicações perioperatórias e hipertensão pulmonar persistente devido à endarterectomia incompleta ou vasculopatia secundária são problemas típicos após o procedimento.^2^ Uma metanálise mostrou que a eficácia da terapia medicamentosa para HPTEP grave é limitada, e muitos pacientes não chegam ter a artéria pulmonar (AP) suficientemente reduzida, mesmo havendo uma melhora marginal da tolerância ao exercício.^3^ A APB percutânea foi reportada pela primeira vez em 2001, mas sua segurança não foi comprovada na época.^4^ Recentemente, há apenas uma década, a hipertensão pulmonar foi associada pela primeira vez a um prognóstico ruim. Felizmente, os tratamentos melhoraram drasticamente desde então, particularmente para pacientes com HPTEC. A APB ainda é uma estratégia desafiadora, pois tem a limitação de um operador especializado e instalação específica, mas seus resultados têm melhorado.^5^

## Relato de Caso

**Figura 1:**
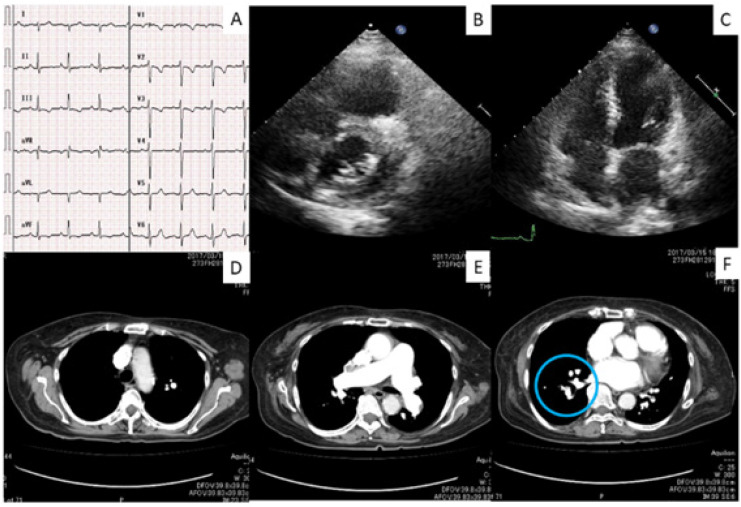
Exames fisiológicos e radiológicos antes da angioplastia pulmonar com balão. A) Eletrocardiograma na admissão mostrava S-I e T-III. B e C) Ecocardiografia transtorácica (fase diastólica final) mostrando insuficiência cardíaca direita (B) na admissão e insuficiência cardíaca direita normalizada dois anos após a APB (C). D-F) Tomografia computadorizada de tórax com contraste mostrando a área avascular no lobo superior direito (D), sem evidência de embolia pulmonar aguda no tronco da artéria pulmonar (E), e achado semelhante a uma teia no ramo da artéria pulmonar direita 8 (círculo azul em F).

Paciente do sexo feminino, 76 anos, 1,45 m de altura, 40 kg, apresentou-se ao serviço com história de dispneia ao esforço havia três meses. Sem histórico de trombose venosa profunda ou embolia pulmonar aguda. Uma semana antes da internação, a dispneia piorou (classe IV da New York Heart Association), a paciente desenvolveu edema nas pernas e tornou-se incapaz de andar. Na admissão, tinha pressão arterial de 210/95 mmHg, frequência cardíaca de 85 bpm, SpO_2_ de 80% (ar ambiente) e frequência respiratória de 32 respirações por minuto. O eletrocardiograma revelou ritmo sinusal e hipertrofia ventricular direita (HVD) (Figura 1A). Os exames laboratoriais mostraram creatinina 0,84 mg/dl, hemoglobina 17,4 g/dl e peptídeo natriurético cerebral (BNP) 1000 pg/ml. Nenhuma evidência de doença vascular do colágeno foi encontrada. A ecocardiografia transtorácica revelou insuficiência cardíaca direita [dilatação do átrio e ventrículo direitos; pressão sistólica da AP estimada em 58 mmHg; 6% de alteração da área fracionária do ventrículo direito; derrame pericárdico] com contração ventricular esquerda preservada (fração de ejeção: 65%) (Figura 1B).

**Figura 2:**
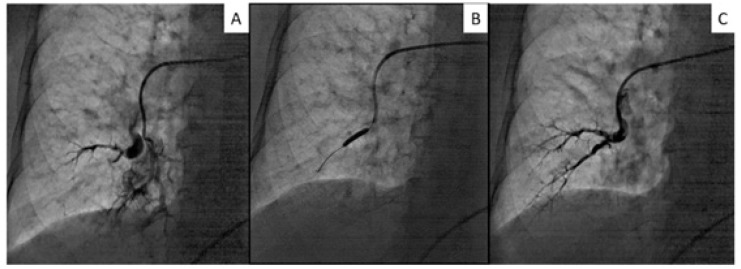
Angiografia da artéria pulmonar representativa em angioplastia pulmonar com balão sistemática. A: Artéria pulmonar direita ocluída (ramo 8) detectada por angiografia seletiva. B: APB com cateter balão semicomplacente de 3,0 mm. C: Angiografia pulmonar seletiva final após APB sistemática com cateteres balão semicomplacente de 2,0 e 3,0 mm.

**Figura 3:**
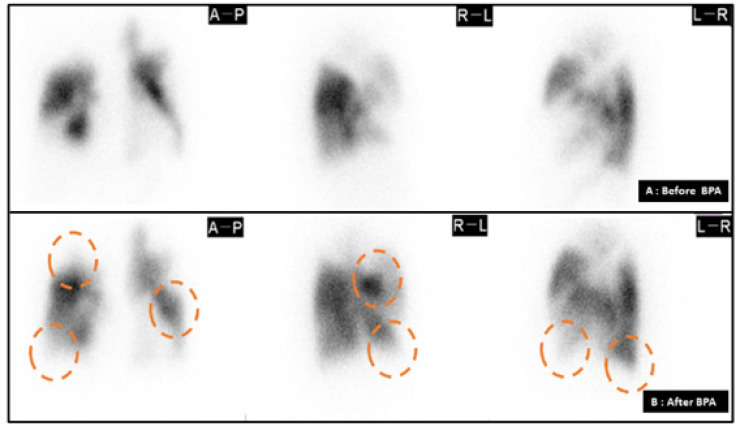
Cintilografia de perfusão pulmonar antes e depois da angioplastia pulmonar sistemática com balão. A e B) Cintilografia de perfusão pulmonar antes (A) e depois (B) da APB. Os círculos laranja mostram áreas de perfusão melhoradas.

Foi submetida a um cateterismo cardíaco direito, que revelou pressão da AP a 60/38 (47) mmHg, pressão de oclusão da artéria pulmonar expiratória final (PAWP) de 6 mmHg, índice cardíaco de 2,17 L/min e índice de resistência vascular pulmonar de 18,89 unidades Wood•m^2^. A angiografia coronária mostrou-se normal. Uma tomografia computadorizada de tórax com contraste não mostrou evidências de embolia pulmonar aguda (Figura 1D-F). Angiografia da AP revelou lesões em teias e de oclusão total e subtotal em artérias segmentares a subsegmentares bilaterais (Figura 2A). Uma cintilografia de perfusão pulmonar mostrou múltiplas lesões bilaterais (Figura 3A).

A paciente recebeu infusões contínuas de heparina e dobutamina em baixas doses durante um mês; no entanto, sua condição não melhorou e ela foi diagnosticada com HPTEC. Até três meses antes da internação, realizava todas as atividades de forma independente, porém, por ser idosa e frágil, referiu estar completamente acamada por uma semana antes da internação. Sua fragilidade (escala clínica canadense 8) e múltiplas lesões distais fizeram dela uma candidata não favorável à cirurgia, então a equipe médica decidiu, após discussões com um cirurgião cardíaco, realizar APB por se tratar de um procedimento menos invasivo e de menor risco.

Os vasos-alvo foram as artérias pulmonares direitas (A1, A2, A3, A5, A7 e A8) e esquerdas (A 3, A4, A6 e A10). O procedimento foi realizado com um sistema de cateter-guia de 0,014 polegadas, semelhante a uma intervenção coronária percutânea. Um cateter-guia 6 French Amplatz esquerdo foi inserido no sentido de um ramo da AP através da veia femoral direita. Foi realizada com dois tipos de cateter-guia de baixo peso (B-pahm 0,6g, Japan Lifeline, Tóquio, Japão) (Chevalier Floppy 2g, FMD, Tóquio, Japão), com suporte de um cateter balão (CB). No primeiro procedimento, a APB foi iniciada pela parte anterior (A3 direita e A5 A3 esquerda) usando um CB semicomplacente de 2,0 mm (Ikazuchi PAD, Kaneka, Osaka, Japão). Auma APB adicional usando CB de 2,0 mm foi realizada na porção póstero-lateral (A3, A5, A7 e A8 direita, A4, A6 e A10 esquerda) um mês depois. Dois meses mais tarde, expandimos todas as artérias pré-dilatadas com um CB de 3,0 mm (Figura 2B e 2C). A APB foi concluída sem orientação de imagem intravascular e sem complicações, como lesão pulmonar e hemoptise.

Não conseguimos abrir as artérias completamente ocluídas (A 1 e A2 direita); entretanto, a pressão da AP diminuiu para 42/16 (26) mmHg imediatamente após a APB final, e a pressão da PA média finalmente melhorou para 20 mmHg sem hipoxemia (SpO_2_ 96%, ar ambiente). Uma nova cintilografia de perfusão pulmonar mostrou melhora da perfusão, com circulação pulmonar adequada em dois terços do leito vascular total da AP (Figura 3B). Ela passou a deambular de forma independente e teve alta hospitalar. Na alta, sua distância de caminhada de seis minutos era de 236 m. Foi prescrito oxigênio suplementar, anticoagulantes e um diurético de baixa dosagem. O BNP diminuiu para 64 pg/ml. Um ecocardiograma mostrou que as dilatações atrial e ventricular direita haviam se normalizado (Figura 1C). Desde então, a paciente manteve um bom quadro clínico ao longo de dois anos, mas um monitoramento adicional é recomendado.

## Discussão

Nossa paciente apresentou HPTEC grave inoperável, sem trombos na parte principal das artérias pulmonares à tomografia computadorizada. Angiografia pulmonar mostrou artérias pulmonares segmentares ocluídas (A 1, 2, 8 direita e A 10 esquerda) e lesões em teia em outras artérias segmentares ou subsegmentares.^6^

Neste caso, realizamos três sessões separadas de APB; no entanto, sessões mais espaçadas podem ser aceitáveis, dependendo da fragilidade e do estado geral do paciente, a fim de evitar lesão pulmonar (como sangramento) por dano a vaso pulmonar.^1^^,^^7^ Também consideramos incompatibilidade ventilação-perfusão e iniciamos o procedimento pela porção anterior para melhorar a hipoxemia. Lesões oclusivas são um preditor de complicações relacionadas à APB.^8^ Em seguida, prosseguimos ao tratamento das artérias obstruídas de forma incompleta, uma vez que as complicações relacionadas ao procedimento podem piorar criticamente a condição hemodinâmica e respiratória da paciente. Foi selecionado um CB pequeno (de 2,0 mm) para evitar o fluxo sanguíneo de alta pressão e, em seguida, as múltiplas artérias-alvo foram dilatadas.

Após a dilatação por fluxo sanguíneo de entrada por dois meses após a APB inicial, todas as artérias tratadas foram dilatadas com um CB de 3,0 mm, de acordo com o diâmetro anatômico da AP. Um relato recente descreveu que pacientes com HPTEC apresentam aumento na rigidez arterial.^9^ A hipertensão arterial sistêmica é incomum na HPTEC, mas, no presente caso, foi normalizada após a APB.

Grupos japoneses relataram resultados melhores em longo prazo associados à APB para pacientes com HPTEC e lesões distais.^10^ Mais estudos observacionais prospectivos e ensaios clínicos randomizados são necessários para comparar a APB e a terapia medicamentosa em pacientes com HPTEC inoperável, determinando assim a eficácia do procedimento.

## Conclusão

A APB sistemática, um tratamento de AP anteroposterior com dois cateteres-balão com diâmetros diferentes (2,0 mm e 3,0 mm), pode ser realizada com segurança, mesmo em pacientes inoperáveis e em condições físicas severas. Hoje em dia, a APB pode não ser o último recurso, mas sim o tratamento de primeira escolha para a população com HPTEC inoperável.
