# Supplementary material for: Angioplastia Pulmonar com Balão Percutâneo Sistemática com Estadiamento em Hipertensão Pulmonar Tromboembólica Crônica Inoperável Grave
Source: Arq Bras Cardiol. 2021 Feb 2;116(2 Suppl 1):21–4. [Article in Portuguese] doi: 10.36660/abc.20190717 (PMC8118630; doi:10.36660/abc.20190717)
Supplement: Translation

# Translation: Systematic Staged Percutaneous Balloon Pulmonary Angioplasty in Severe Inoperable Chronic Thromboembolic Pulmonary Hypertension

**Authors:** Kazuhiro Dan, Akira Shionoda, Hiromi Matsubara

**Affiliations:** 1 Ichinomiya Japan Ichinomiya Nishi Hospital, Ichinomiya - Japan; 2 Okayama Japan Okayama Medical Center, Okayama - Japan

**Keywords:** Angioplasty, Balloon, Pulmonary, Hypertension, Pulmonary, Pulmonary Embolism, Aging, Fragility

## Introduction

The treatment for chronic thromboembolic pulmonary hypertension (CTEPH) is limited to pulmonary endarterectomy (PEA), drug therapy, and percutaneous balloon pulmonary angioplasty (BPA).^1^ The gold-standard treatment is PEA, and patients with CTEPH and proximal lesions are generally good surgical candidates. Perioperative complications and persistent pulmonary hypertension due to incomplete endarterectomy or secondary vasculopathy are typical problems after the procedure.^2^ A meta-analysis has shown that the efficacy of drug therapy for severe CTEPH is limited, and many patients do not achieve sufficiently reduced pulmonary artery (PA) pressure, even if exercise tolerance is marginally improved.^3^ Percutaneous BPA was first reported in 2001, but its safety was not proven at that time.^4^ As recently as one decade ago, pulmonary hypertension was first associated with poor prognosis. Fortunately, treatments for pulmonary hypertension have dramatically improved ever since, particularly for patients with CTEPH. BPA is still a challenging strategy, limited to a specific operator and facility, but its results have been improving.^5^

## Case Report

A 76-year-old female patient (height 1.45 m, weight 40 kg) presented to the service with a three-month history of dyspnea on exertion. She had no history of deep vein thrombosis or acute pulmonary embolism. One week before admission, her dyspnea worsened (New York Heart Association class IV), she developed leg edema and became unable to walk. On admission, her blood pressure was 210/95 mmHg, heart rate was 85 bpm, SpO_2_ 80% (room air), and respiratory rate was 32 breaths per minute. An electrocardiogram showed sinus rhythm and right ventricular hypertrophy (RVH) (Figure 1A). Laboratory exams showed creatinine level at 0.84 mg/dl, hemoglobin level at 17.4 g/dl, and brain natriuretic peptide (BNP) level at 1000 pg/ml. No evidence of collagen vascular disease was noted. A transthoracic echocardiography revealed right heart failure [right atrium and ventricle dilatation; estimated systolic PA pressure of 58 mmHg; 6% right ventricular fractional area change; pericardial effusion] with preserved left ventricular contraction (ejection fraction: 65%) (Figure 1B).

**Figure 1:** Physiological and radiological examinations before balloon pulmonary angioplasty. A) Electrocardiogram on admission showed S-I and T-III. B and C) Transthoracic echocardiography (end diastolic phase) showing right heart failure (B) upon admission, and right heart failure normalized two year after BPA (C). D-F) Contrast-enhanced chest computed tomography showing the avascular area in the right upper lobe (D), no evidence of acute pulmonary embolism in main pulmonary artery (E), and web-like finding in the right pulmonary artery branch 8 (blue circle in F).

She underwent right heart catheterization, which revealed PA pressure of 60/38 (47) mmHg, pulmonary artery wedge pressure (PAWP) of 6 mmHg, cardiac index of 2.17 L/min and pulmonary vascular resistance index of 18.89 Wood unit•m^2^. Coronary angiography was normal. Contrast-enhanced chest computed tomography scan showed no evidence of acute pulmonary embolism (Figure 1D-F). PA angiography revealed webs, and subtotal and total occlusion lesions in bilateral segmental to subsegmental pulmonary arteries (Figure 2A). Lung perfusion scintigraphy showed multiple bilateral defects (Figure 3A).

**Figure 2:** A representative pulmonary artery angiography in systematic staged balloon pulmonary angioplasty. A: Occluded right pulmonary artery (branch 8) detected by selective angiography. B: BPA with a 3.0-mm semicompliant balloon catheter. C: Final selective pulmonary angiography after systematic staged BPA with 2.0- and 3.0-mm semicompliant balloon catheters.

**Figure 3:** Lung perfusion scintigraphy before and after systematic balloon pulmonary angioplasty. A and B) Lung perfusion scintigraphy prior to (A) and after (B) BPA. Orange circles show improved perfusion areas.

The patient was put on continuous infusions of heparin and low-dose dobutamine over a month; however, her condition did not improve and she was diagnosed with CTEPH. Until three months before admission, she independently performed all activities, but being elderly and frail, she reported being completely bedridden for one week prior to admission. Her frailty (Canadian clinical scale 8) and multiple distal lesions rendered her a poor operative candidate, so the medical staff decided to perform BPA, as this is a less-invasive and lower-risk treatment, after discussions with a cardiac surgeon.

The target vessels were right (A1, A2, A3, A5, A7, and A8) and left pulmonary arteries (A 3, A4, A6, and A10). The procedure was performed using a 0.014-inch guidewire system, similar to a percutaneous coronary intervention. A 6 French Amplatz left catheter was directed into a branch of the PA via the right femoral vein. The wiring was performed with two kinds of low-weight guidewire (B-pahm 0.6g, Japan Lifeline, Tokyo, Japan) (Chevalier Floppy 2g, FMD, Tokyo, Japan), supported by a balloon catheter (BC). In the first procedure, BPA was initiated from the anterior part (Right A3 and A5 Left A3.) using a 2.0-mm semicompliant BC (Ikazuchi PAD, Kaneka, Osaka, Japan). Additional BPA using a 2.0-mm BC was performed on the posterolateral portion (Right A3, A5, A7 and A8. Left A4, A6 and A10.) one month later. Two months after that, we expanded all pre-dilated arteries using a 3.0-mm BC (Figure 2B and 2C). BPA was completed without intravascular imaging guidance and without complications such as lung injury and hemoptysis. We were not able to open the completely occluded arteries (Right A 1 and A2); however, her PA pressure decreased to 42/16 (26) mmHg immediately after the final BPA, and the mean PA pressure finally improved to 20 mmHg without hypoxemia (SpO_2_ 96%, room air). A new lung perfusion scintigraphy showed improved perfusion, with adequate pulmonary circulation over two-thirds of the total PA vascular bed (Figure 3B). She could ambulate independently and was discharged from the hospital. On discharge, her six-minute walking distance was 236 m. She was prescribed supplemental oxygen, anticoagulants, and a low-dose diuretic drug. Her BNP decreased to 64 pg/ml. The right atrial and ventricular dilatations normalized on echocardiography (Figure 1C). Ever since, the patient has maintained a good clinical picture over the course of two years, but further monitoring is recommended.

## Discussion

Our patient presented with severe inoperable CTEPH without thrombi at the main part of pulmonary arteries on computed tomography scan. Pulmonary angiography showed occluded pulmonary segmental arteries (right A 1, 2, 8 and left A 10) and arterial webbing in other segmental or subsegmental arteries.^6^

In this case, we performed three separate BPA sessions; however, more spaced sessions may be acceptable depending on the frailty and general condition of the patient in order to avoid lung injury (such as bleeding) due to pulmonary vessel injury.^1^^,^^7^ We also considered ventilation-perfusion mismatch and started the procedure from the anterior part to improve hypoxemia. Occlusive lesions are a predictor of BPA-related complications.^8^ We then proceeded with treating incompletely occluded arteries, once BPA-related complications may critically worsen the patient's hemodynamics and respiratory condition. A small BC (2.0 mm) was selected to avoid high-pressure incoming blood flow and then the multiple target arteries were dilated.

After dilation by incoming blood flow for two months from initial BPA, all treated arteries were dilated using a 3.0-mm BC, as per the anatomical diameter of PA. A recent report described that patients with CTEPH present increased arterial stiffness.^9^ High systemic blood pressure is uncommon in CTEPH, but, in the present case, it was normalized after BPA.

Japanese groups have reported improved long-term outcomes associated with BPA for patients with CTEPH and distal lesions.^10^ Further prospective observational studies and randomized controlled trials are required to compare BPA and drug therapy in patients with inoperable CTEPH, thus determining the efficacy of the procedure.

## Conclusion

Systematic staged BPA, a treatment from anterior to posterior PA by two balloon catheters with different diameters (2.0 mm and 3.0 mm), can be safely performed even in inoperable patients with severe physical conditions. Nowadays, BPA may not be the last resort, but rather the first-choice treatment for the inoperable CTEPH population.
